# Validity of two common asthma-specific quality of life questionnaires: Juniper mini asthma quality of life questionnaire and Sydney asthma quality of life questionnaire

**DOI:** 10.1186/1477-7525-10-97

**Published:** 2012-08-20

**Authors:** Christian Joachim Apfelbacher, Christina Jones, Matthew Hankins, Helen Smith

**Affiliations:** 1 Division of Public Health and Primary Care, Brighton and Sussex Medical School, Brighton, United Kingdom; 2 Medical Sociology, Institute of Epidemiology and Preventive Medicine, University of Regensburg, Regensburg, Germany; 3 Faculty of Health Sciences, University of Southampton, Southampton, UK

## Abstract

**Background:**

This study explored the psychometric properties (internal consistency, construct validity, discriminative ability) of the Juniper Mini Asthma Quality of Life Questionnaire (Mini AQLQ-J) and the Sydney Asthma Quality of Life Questionnaire (AQLQ-S).

**Methods:**

One hundred fourty-six adults (18–45 years) with asthma requiring regular inhaled corticosteroids were recruited to a trial of written emotional disclosure. Correlational analyses were performed to understand the relationship of the two measures with each other, with symptoms, lung function, asthma control, asthma bother and generic quality of life. Median quality of life scores were compared according to gender, health care usage and levels of asthma severity.

**Results:**

AQLQ-J and AQLQ-S total scores correlated strongly with each other (*rho* = −0.80) and moderately with the EuroQol Current Health Status Scale (AQLQ-J: *rho* = 0.35; AQLQ-S: *rho* = −0.40). Domain score correlations between AQLQ-J and AQLQ-S were mostly moderate (0.50 < *rho* < 0.80).

Both QoL measures were significantly correlated with symptom score. Correlations with the symptom score asthma module (AQLQ-J: *rho* = −0.69; AQLQ-S: *rho* = 0.50) were stronger compared with the total symptom score and the symptom score rhinitis module (AQLQ-J: *rho* = −0.41; AQLQ-S: *rho* =0.31).

Neither QoL measure was significantly correlated with FEV1, % predicted at the total or the domain level.

Total scores of both measures were significantly correlated with subjective asthma control (AQLQ-J: *rho* = 0.68; AQLQ-S: *rho* = −0.61) and asthma bother (AQLQ-J: *rho* = −0.73; AQLQ-M: *rho* = 0.73).

Total AQLQ-J score and total AQLQ-S score were significantly associated with perceived asthma severity (AQLQ-J: p=0.004, AQLQ-S: p=0.002) and having visited a GP in the past four months (AQLQ-J: p=0.003, AQLQ-S: p=0.002).

**Conclusions:**

This study provides further evidence for the validity of the AQLQ-J and the AQLQ-S in a British population of adult patients with asthma managed in primary care. Correlations with lung function parameters were weak or absent. Correlations with generic quality of life were moderate, those with asthma symptoms, asthma control and asthma bother were strong. Both measures are able to discriminate between levels of asthma severity and health care usage.

## Background

Patient reported outcome measures (PROMs) have gained importance in clinical trials, epidemiological surveys, audit and clinical practice. ‘PROM’ is an umbrella term proposed by the US Food and Drug Administration (FDA) denoting the “measurement of any aspect of a patient’s health status that comes directly from the patient (i.e. without the interpretation of the patient’s responses by a physician or anyone else)” [1]. PROMs are a means of quantifying qualitative information requiring careful consideration in their development [2].

Health-related quality of life (HrQoL) is among the most important patient assessed health outcomes but is also referred to as health status, perceived health or simply ‘quality of life’ [3]. No single concept has been universally adopted [1,4,5].

For adults with asthma, a variety of health related quality of life (HrQoL) measures are in use. A recent review identified six commonly used questionnaires and concluded that the measures differ substantially in a number of aspects (conceptual and measurement model, reliability, validity, interpretability, burden of completion, administration format, validated translations) [6]. Among these measures were the Sydney Asthma Quality of Life Questionnaire (AQLQ-S) [7,8] and the Juniper Asthma Quality of Life (AQLQ-J) Questionnaire [9,10].

The validation of a PROM is a continuous process as evidence needs to accumulate to increase the confidence in the reliability and validity of a measure. Estimates of reliability and validity are always sample-dependent (i.e. the coefficients obtained are expected to vary from sample to sample) [11], hence it is important to publish the respective coefficients so a clear picture of the performance of the questionnaires can be built up. We therefore studied the internal consistency and validity (construct validity and discriminative ability) of the AQLQ-S and the AQLQ-J (mini version).

Furthermore, few studies have been conducted which look at quality of life measures used for asthma in a comparative manner [12-14]. This is important to get a sense of the relative validity and conceptual structure of PROMs which claim to measure the same thing. As a secondary objective, we therefore compared the psychometric properties of the AQLQ-S and the Mini AQLQ-J.

## Methods

First, the study population was characterised by means of descriptive statistics. Second, measures of internal consistency were calculated for the mini AQLQ-J and the AQLQ-S.

Third, we investigated how the two measures related to:

· each other

· symptom score

· lung function

· asthma control

· asthma bother

· generic quality of life

Finally, the discriminative ability of the two asthma-specific measures with respect to age, gender, health care utilisation (GP visits) and perceived asthma severity was examined.

### Data source

Data were drawn from the baseline assessment of a double blind randomised controlled trial (RCT) investigating the effects of a written emotional disclosure (WED) intervention in adult patients with asthma in the UK. One hundred fourty-six adults (18–45 years) with a diagnosis of asthma requiring regular inhaled corticosteroids were recruited into this trial. Participants were allocated to receive either WED or non-emotional writing instructions and asked to write for 20 minutes over three consecutive days. Spirometry, health care utilisation, asthma-specific and generic quality of life, rhinoconjunctivitis and asthma symptoms, subjective asthma control and asthma bother were documented. The participants’ lung function was measured using a Vitalograph Micro spirometer. The remaining constructs were assessed using self-administered questionnaires (the questionnaires were sent to participants and completed prior to the visit by the researcher, i.e. prior to the administration of the intervention). The questionnaires were administered in the same order each time: Rhinoconjunctivitis and Asthma Symptom Score, Asthma Control Test, Mini AQLQ-J, AQLQ-S, Profile of Mood States, EuroQoL Current Health status, Significant Other Scale and Asthma Bother Profile. It must be noted that we used the mini version of the AQLQ-J because this and not the original version was included in the randomised controlled trial (RCT) from which the data was drawn. The Profile of Mood States and the Significant Other Scale were not considered for this paper.

The trial was registered with http://www.controlled-trials.com, registration number ISRCTN82986307. Ethical approval was obtained from the Brighton and Mid Sussex Research Ethics Committee (reference number 04/Q1907/91).

### Quality of life measures

The AQLQ-J was developed in Canada and comprises 32 items in four domains with a two-week recall period [9,10]. Higher ratings denote less impairment (better quality of life). Later, a shorter and simpler questionnaire with 15 items in the same domains was developed (Mini AQLQ-J) [15]. There are five items in the domain ‘Symptoms’, four items in the domain ‘Activity Limitations’, three items in the domain ‘Emotional Function’ and three items in the domain ‘Environmental Stimuli’. Higher scores indicate better quality of life. This questionnaire showed good measurement properties, but they were not quite as strong as for the original AQLQ-J [15]. The minimal important difference of quality of life score per item has been reported to be very close to 0.5 (range 0.42-0.58) for the original AQLQ-J [16].

The AQLQ-S was developed in Australia and comprises 20 items with a four-week recall period [7,8]. Lower ratings indicate less impairment (better quality of life). Items are grouped into four domains (breathlessness: five items, mood: five items, social: seven items, concerns: three items). The content of the questionnaires is illustrated in Table 1. For the AQLQ-S, a minimal important difference has not been reported.

**Table 1:** Number of items and content of the AQLQ-J and the AQLQ-S

**Domain**	**Number of items**	**Content**
AQLQ-J symptoms	5	Feeling short of breath as a result of asthma, feeling bothered by coughing, experiencing a feeling of chest tightness or chest heaviness, having difficulty getting a good night’s sleep as a result of asthma, experiencing a wheeze in the chest
AQLQ-S breathlessness	5	Having been troubled by episodes of shortness of breath, having been troubled by wheezing attacks, having been troubled by tightness in the chest, having been restricted in walking down the streets or level ground or doing light housework because of asthma, having been restricted in walking up hills or doing heavy housework because of asthma
AQLQ-J environment	3	Feeling bothered by or having to avoid dust in the environment, feeling bothered by or having to avoid cigarette smoke in the environment, feeling bothered or having to avoid going outside because of weather or air pollution
AQLQ-S concerns	3	Having been worried about asthma shortening the life, having felt dependent on asthma sprays, having been worried about present or future life because of asthma
AQLQ-J emotions	3	Feeling frustrated as a result of asthma, feeling afraid of not having asthma medication available, feeling concerned about having asthma
AQLQ-S mood	3	Having felt tired or a general lack of energy, having been unable to sleep at night, having felt sad or depressed, having felt frustrated with oneself, having felt anxious, under tension or stressed
AQLQ-J activities	4	Having been limited in doing strenuous activities (such as hurrying, exercising, running up chairs, sports) as a result of asthma, having been limited in doing moderate activities (such as walking, housework, gardening, shopping, climbing stairs) as a result of asthma, having been limited in doing social activities (such as talking, playing with pets/children, visiting friends/relatives), having been limited in doing work-related activities* (tasks you have to do at work)
AQLQ-S social	7	Having felt that asthma is preventing one from achieving what one wants in life, asthma having interfered with one’s social life, having been limited in going to certain places because they are bad for one’s asthma, having been limited in going to certain places because of having been afraid if getting an asthma attack and not being able to get help, having felt generally restricted, having been restricted in the sports, hobbies, or other recreations one can engage in because of one’s asthma, having felt asthma is controlling one’s life

*these should be activities which one has to do most days if not employed or self-employed.

The EuroQoL Current Health Status Scale (CHS) is a generic, preference-based measure of health status and consists of five items (mobility, self-care, usual activities, pain/discomfort, anxiety/depression) [17]. Higher scores indicate better quality of life.

### Lung function measurement

The following parameters were measured in spirometry: forced vital capacity (FVC) in litres [l], forced expiratory volume in one second (FEV1) in litres [l], peak expiratory flow (PEF) in litres per minute [l/min]. The European Coal and Steel Community (ECSC) prediction equations were used to calculate what participant’s optimum lung function should be based on age, height and gender [18,19]. This reading was used with FEV1 scores to determine FEV1% predicted scores.

### Rhinoconjunctivitis and Asthma symptom score

The Rhinoconjunctivitis and Asthma Symptom Score has 21 items which are rated on a five point Likert scale [20,21]. Patients are asked how much they have been disturbed by symptoms during the last week. The rhinitis module of the score asks about symptoms of irritation, congestion and discharge in the eyes, the nose and the sinuses. The asthma module asks about daytime and nighttime symptoms of cough, wheeze, sputum production and shortness of breath.

### Asthma Control Test (ACT)

The Asthma Control Test (ACT) consists of five questions pertaining to the past 4 weeks [22,23]. The brief questionnaire assesses asthma symptoms (daytime and nocturnal), use of rescue medication, and the effect of asthma on daily functioning. The total score is obtained by summing the scores for each item and ranges from 5 (poor control of asthma) to 25 (complete control of asthma).

### Asthma Bother Profile (ABP)

The Asthma Bother Profile (ABP) is a 15 item measure of asthma distress [24]. Patients are asked how much their asthma bothers them in the different areas of their life and they are given six response options (no bother, minor irritation, slight bother, moderate bother, a lot of bother, makes my life a misery) and one ‘not applicable’ option. Higher scores indicate greater distress.

### Statistical analysis

To describe the characteristics of the sample, counts and percent were calculated for categorical variables and means with standard deviations (SD) were calculated for continuous variables.

In order to analyse the distribution of domain and total scores for the two asthma-specific quality of life measures mean, median, standard deviation (SD), percentage of participants with missing items, observed range, the percentage of participants with the worst possible score (‘floor’) and percentage of participants with the best possible score (‘ceiling’) were calculated. Standardized Cronbach’s alpha coefficients were computed as a measure of internal consistency [25].

Correlational analyses were performed to analyse the relationship between scores where appropriate. Spearman rank-order correlations, non-parametric measures of association based on the ranks of the data values, were calculated because the scores of the measures of interest (AQLQ-J and AQLQ-S) were not normally distributed.

In order to analyse the relationship between the domain and total scores of the AQLQ-J and the AQLQ-S, a correlation matrix was computed. Correlations were considered as absent if rho < 0.20, poor if rho = 0.20 <= rho < = 0.34, moderate if rho = 0.35 < = rho < = 0.50 and strong if rho > 0.50 [26]. In order to compare scores between categories (gender, GP visits, perceived severity), non-parametric tests were used (Wilcoxon rank sum two sample test for comparison between two groups and Kruskal-Wallis test for comparison between more than two groups). Missing data in the questionnaires were dealt with by imputing mean values where more than half of the responses to a subscale were present.

All analyses were performed using SAS 9.2 for Windows.

## Results

### Patient characteristics

One hundred fourty-six people with asthma (average disease duration: 21.4 years (*SD*: 11.5 years)), aged 18–45 years and with a mean age of 36.1 years (*SD*: 7.0 years), participated in the study. Most of the participants were female (76.7%), in employment (82.2%) and of white ethnicity (97.3%). Their asthma was perceived as mild by 48.0%, as moderate by 45.9% and as severe by 3.4%. About one quarter (25.3%) of all patients reported consulting their general practitioner because of their asthma during the past four months (excluding asthma review appointments), 3.4% reported they had visited Accident & Emergency (A&E) because of their asthma during the past four months but none reported having been admitted to hospital in the same time period.

The distribution of the questionnaire scores is displayed in Table 2. The mean AQLQ-J score was 5.4 (*SD:* 1.0, minimum 1.7, maximum 7.0), the mean AQLQ-S was 0.8 (*SD* 0.6, minimum 0.1, maximum 3.4). About one quarter of the responses in the ‘activity limitation’ domain of the AQLQ-J and the ‘social’ domain of the AQLQ-S were the best possible scores (ceiling effect).

**Table 2:** Distribution of scores and internal consistency of AQLQ-J and AQLQ-S

	**AQLQ-J**					**AQLQ-S**				
	**Symptoms**	**Environmental Stimuli**	**Emotional Function**	**Activity Limitation**	**Total**	**Breathlessness**	**Mood**	**Social**	**Concerns**	**Total**
Items [n]	5	3	3	4	15	5	5	7	3	20
Mean	5.06	5.38	5.18	5.97	5.39	0.83	0.99	0.52	0.76	0.80
Median	5.20	5.67	5.33	6.25	5.60	0.60	0.80	0.29	0.57	0.70
SD	1.15	1.24	1.48	1.12	1.04	0.60	0.76	0.68	0.66	0.58
Participants with missing items [%]	4.79	4.79	5.48	5.48	6.16	5.48	5.48	4.79	5.48	5.48
Theoretical range	1-7	1-7	1-7	1-7	1-7	0-4	0-4	0-4	0-4	0-4
Observed range	1.80-7.00	1.33-7.00	1.00-7.00	1.50-7.00	1.67-7.00	0-3.00	0-3.80	0-4.00	0-3.57	0.05-3.35
‘Floor’ [%]^*^	0	0	1.45	0	0	0	0	0	0	0
‘Ceiling’ [%]^**^	1.44	10.79	10.87	25.36	0.73	5.80	10.90	26.62	12.32	0
Cronbach’s alpha	0.85 (N=139)	0.61 (N=139)	0.82 (N=138)	0.89 (N=138)	0.92 (N=137)	0.83 (N=138)	0.85 (N=138)	0.91 (N=139)	0.77 (N=139)	0.93 (N=138)

*Percentage of participants with worst possible score.

**Percentage of patients with best possible score.

Mean rhinoconjunctivitis and asthma symptom score was 45.2 (*SD*: 13.9, median: 44.5, range: 20.0-89.0, *N* = 136). For the asthma module, mean symptom score was 20.4 (*SD*: 7.6, median: 19.0, range: 9.0 - 42.0, *N* = 137) and for the rhinitis module, mean symptom score was 24.8 (*SD*: 8.1, median: 25.0, range: 11.0-48.0, *N* = 136).

The following spirometry results were observed (*N* = 145): mean FVC was 3.72 l (*SD* 1.03, range: 1.37-7.35), mean FEV1 was 2.82 l (*SD* 0.77, range: 0.74-4.95), mean FEV1, % predicted was 87.5% (*SD*: 20.5, range: 24.8 - 167.3) and mean PEF was 427.4 l/min (*SD*: 132.2, range: 117.0-847.0).

### Internal consistency

Cronbach’s alpha values for the total and the domain scores of both QoL measures are also displayed in Table 2.

For the subscales of the AQLQ-J, all Cronbach’s alpha values were > = 0.61 and for the subscales of the AQLQ-S, all Cronbach’s alpha values were > = 0.83. Cronbach’s alpha was 0.92 for the total AQLQ-J score and 0.93 for the total AQLQ-S score.

### Correlation of AQLQ-J and AQLQ-S

A correlation matrix of the two measures is shown in Table 3. The total scores of the AQLQ-J and AQLQ-S correlated strongly with each other (*rho* = −0.80, p < 0.0001). The domain scores of the AQLQ-J and AQLQ-S showed weaker, but still strong (0.50 < = *rho* < = 0.80) correlations with each other. The only exception was the ‘mood’ domain of the AQLQ-S which correlated moderately (*rho* < 0.5) with the ‘environmental stimuli’, the ‘emotional function’ and the ‘activity limitation’ domain of the AQLQ-J.

**Table 3:** Relationship of domain and total scores of AQLQ-J and AQLQ-S (correlation matrix)

	**AQLQ-J**					**AQLQ-S**				
	**Symptoms**	**Environmental Stimuli**	**Emotional Function**	**Activity Limitation**	**Total**	**Breathlessness**	**Mood**	**Social**	**Concerns**	**Total**
**AQLQ-J**										
Symptoms	-	0.54	0.68	0.60	0.90					
Environmental Stimuli		-	0.52	0.47	0.73					
Emotional Function		0.52	-		0.82					
Activity Limitation		0.47	0.51	-	0.78					
Total					-					
**AQLQ-S**										
Breathlessness	-0.74	-0.42	-0.54	-0.61	-0.72	-				
Mood	-0.57	-0.36	-0.46	-0.42	-0.58	0.57	-			
Social	-0.52	-0.54	-0.53	-0.70	-0.68	0.56	0.41	-		
Concerns	-0.59	-0.45	-0.65	-0.53	-0.68	0.59	0.61	0.68	-	
Total	-0.73	-0.52	-0.66	-0.66	-0.80	0.81	0.80	0.76	0.88	

Spearman’s rank correlation coefficients, all correlation coefficients showed p values < 0.0001.

Correlations between AQLQ-J and AQLQ-S scores are negative because the respective scores run in opposite directions.

### Correlation of AQLQ-J and AQLQ-S with Rhinoconjunctivitis and Asthma Symptom Score

Both QoL measures were significantly correlated with the Rhinoconjunctivitis and Asthma Symptom Score, but the correlation of the AQLQ-J with the symptom score was stronger (*rho* = −0.62, *p* < 0.0001, *N* = 134) compared with the correlation of the AQLQ-S (*rho* = 0.46, *p* < 0.0001, *N* = 135). For both QoL measures, correlations with the symptom score asthma module (AQLQ-J: *rho* = −0.69, *p* < 0.0001, *N* = 135; AQLQ-S: *rho* = 0.50, *p* < 0.0001, *N* = 136) were stronger compared with the total symptom score and the symptom score rhinitis module (AQLQ-J: *rho* = −0.41, *p* < 0.0001, *N* = 134; AQLQ-S: *rho* = 0.31, *p* = 0.0002, *N* = 135).

### Correlation of AQLQ-J and AQLQ-S with lung function

As shown in Table 4, the AQLQ-J total score and all domain scores except ‘emotional function’ were significantly correlated with FVC, but only the association with environmental stimuli was of moderate strength. Regarding the AQLQ-S, a significant, but weak correlation with FVC was found for the ‘breathlessness’ domain. The AQLQ-J total score and the domains ‘environmental stimuli’ as well as ‘activity limitation’ showed significant, but weak correlations with FEV1 and PEF.

**Table 4:** Relationship of spirometry parameters with domain and total scores of AQLQ-J and AQLQ-S

	**AQLQ-J**					**AQLQ-S**				
	**Symptoms (N=138)**	**Environmental Stimuli (N=138)**	**Emotional Function (N=137)**	**Activity Limitation (N=137)**	**Total (N=136)**	**Breathlessness (N=137)**	**Mood (N=137)**	**Social (N=138)**	**Concerns (N=137)**	**Total (N=137)**
FVC	0.24 (p=0.005)	0.36 (p<0.0001)	0.17 (p=0.05)	0.33 (p<0.0001)	0.33 (p<0.0001)	-0.22 (p=0.01)	-0.12 (p=0.15)	-0.16 (p=0.05)	-0.11 (p=0.19)	-0.17 (p=0.05)
FEV1	0.15 (p=0.07)	0.20 (p=0.02)	0.08 (p=0.38)	0.23 (p=0.007)	0.20 (p=0.02)	-0.16 (p=0.07)	-0.05 (p=0.56)	-0.07 (p=0.39)	-0.06 (p=0.50)	-0.09 (p=0.31)
PEF	0.13 (p=0.13)	0.26 (p=0.002)	0.12 (p=0.17)	0.32 (p=0.0001)	0.24 (p=0.006)	-0.18 (p=0.04)	-0.09 (p=0.32)	-0.22 (p=0.01)	-0.09 (p=0.32)	-0.14 (p=0.09)
FEV1, % predicted	0.15 (p=0.08)	0.09 (p=0.32)	0.10 (p=0.25)	0.17 (p=0.05)	0.16 (p=0.06)	-0.17 (p=0.05)	-0.01 (p=0.88)	-0.09 (0.31)	-0.10 (p=0.26)	-0.09 (p=0.29)

Spearman’s rank correlation coefficients.

Neither the total score nor any domain score of the AQLQ-S were significantly correlated with FEV1. There were, however, significant correlations of the AQLQ-S domains ‘breathlessness’ and ‘social’ with PEF. The correlation with ‘breathlessness’ qualified as absent, while the correlation with ‘social’ classified as weak. No significant correlation of either QoL measure at total or domain level was observed with FEV1, % predicted.

### Correlation of AQLQ-J and AQLQ-S with asthma control

Both AQLQ-J total score (*rho* = 0.68, *p* < 0.0001, *N* = 136) and AQLQ-S total score (*rho* = −0.61, *p* < 0.0001, *N* = 137) were significantly correlated with asthma control.

### Correlation of AQLQ-J and AQLQ-S with Asthma Bother Profile (ABP)

Both QoL measures correlated significantly with the ABP to the same degree (AQLQ-J: *rho* = −0.73, *p* < 0.0001, *N* = 130; AQLQ-S: *rho* = 0.73, *p* < 0.0001, *N* = 131).

### Correlation of AQLQ-J and AQLQ-S with EuroQoL current health status (CHS)

Both AQLQ-J (*rho* = 0.35, *p* < 0.0001, *N* = 134) and the AQLQ-S (*rho* = −0.40, *p* < 0.0001, *N* = 135) correlated significantly with the EuroQoL Current Health Status Scale (CHS).

### Relationship of AQLQ-J and AQLQ-S to patient characteristics (age, gender, GP visits, perceived asthma severity)

Neither measure correlated with age (*rho* = 0.01, *p* = 0.92, *N* = 137 for the AQLQ-J and *rho* = −0.03, *p* = 0.77, *N* = 138 for the AQLQ-S). As shown in Table 5, there was no significant difference between male and female in total AQLQ-J scores, but a significantly better QoL score was observed in the male group for the domains ‘environmental stimuli’ and ‘activity limitation’. No significant difference between male and female was observed for the AQLQ-S total and in the domains.

**Table 5:** Median score (interquartile range) of AQLQ-J and AQLQ-S according to patient characteristics

	**AQLQ-J**					**AQLQ-S**				
	**Symptoms**	**Environmental Stimuli**	**Emotional Function**	**Activity Limitation**	**Total**	**Breathlessness**	**Mood**	**Social**	**Concerns**	**Total**
**Gender**										
Female	5.20 (4.20-6.00) N=105	5.67 (4.67-6.00) N=105	5.33 (4.00-6.33) N=105	6.25 (5.25-6.75) N=104	5.50 (4.57-6.07) N=104	0.80 (0.40-1.20) N=104	0.80 (0.40-1.40) N=104	0.29 (0.00-0.86) N=105	0.57 (0.29-1.14) N=104	0.70 (0.35-1.10) N=104
Male	5.10 (4.60-6.00) N=34	6.00 (5.33-7.00) N=34	5.67 (4.33-6.33) N=33	6.63 (5.75-7.00) N=34	5.93 (5.27-6.20) N=33	0.60 (0.40-1.00) N=34	0.90 (0.40-1.40 N=34	0.14 (0.00-0.71) N=34	0.64 (0.29-1.00) N=34	0.63 (0.45-0.95) N=34
P value*	0.58	0.003	0.61	0.03	0.10	0.48	0.70	0.39	0.74	0.69
**Health care usage (GP visit) during the past four months**										
Yes	4.20 (3.60-5.20) N=35	5.33 (4.00-6.00) N=35	5.00 (4.00-6.33) N=35	6.00 (4.25-6.75) N=35	4.93 (4.40-5.93) N=35	1.00 (0.60-1.60) N=35	1.20 (0.80-1.80) N=35	0.29 (0.00-1.29) N=35	0.86 (0.43-1.43) N=35	0.90 (0.55-1.55) N=35
No	5.40 (4.60-6.10) N=104	5.67 (4.67-6.33) N=104	5.67 (4.33-6.67) N=103	6.25 (5.75-7.00) N=103	5.77 (5.13-6.20) N=102	0.60 (0.40-1.00) N=103	0.80 (0.40-1.20) N=103	0.29 (0.07-0.71) N=104	0.57 (0.29-1.00) N=103	0.55 (0.35-0.95) N=103
P value*	0.0009	0.05	0.09	0.04	0.003	0.002	0.0003	0.51	0.01	0.002
**Perceived asthma severity**										
No symptoms	6.10 (5.40-6.20) N=4	6.00 (5.50-6.50) N=4	6.00 (5.50-6.67) N=4	6.88 (6.38-7.00) N=4	6.27 (5.73-6.53) N=4	0.30 (0.20-0.50) N=4	0.40 (0.10-0.70) N=4	0.14 (0.07-0.50) N=4	0.00 (0.00-0.43) N=4	0.23 (0.15-0.48) N=4
Mild	5.40 (4.60-6.20) N=67	5.67 (4.67-6.00) N=67	6.00 (4.67-6.67) N=67	6.50 (6.00-7.00) N=66	5.80 (5.20-6.33) N=66	0.60 (0.40-0.80) N=66	0.80 (0.40-1.20) N=66	0.14 (0.00-0.71) N=67	0.43 (0.29-1.00) N=66	0.53 (0.35-0.90) N=66
Moderate	5.00 (3.90-5.80) N=64	5.67 (4.67-6.33) N=64	5.00 (4.00-6.00) N=63	6.25 (5.13-6.75) N=64	5.33 (4.53-5.93) N=63	0.80 (0.60-1.40) N=64	0.98 (0.50-1.40) N=64	0.29 (0.00-0.86) N=64	0.71 (0.43-1.21) N=64	0.73 (0.45-1.28) N=64
Severe	3.50 (2.60-4.10) N=4	4.17 (2.67-5.33) N=4	3.50 (2.17-5.33) N=4	4.50 (3.63-5.88) N=4	4.20 (2.93-4.93) N=4	1.70 (1.30-2.20) N=4	1.70 (1.60-2.80) N=4	1.38 (0.29-2.93) N=4	1.43 (0.71-2.79) N=4	1.58 (1.03-2.65) N=4
P value**	0.003	0.28	0.01	0.05	0.004	0.0002	0.007	0.29	0.008	0.002

*Wilcoxon rank sum two sample test (normal approximation, including continuity correction).

**Kruskal-Wallis test.

Scores were significantly worse for those patients who reported having visited their GP during the past four months for the AQLQ-J total and the ‘symptom’ and ‘activity limitation’ domains. They were also significantly worse for the AQLQ-S total as well as the ‘breathlessness’ , ‘mood’ and ‘concerns’ domains.

The relationship between median quality of life scores and perceived asthma severity was significant for the ‘symptoms’ and ‘emotional function’ domain scores of the AQLQ-J as well as the total AQLQ-J score. The relationship between median quality of life scores and perceived asthma severity was also significant for the ‘breathlessness’, ‘mood’, ‘concerns’ domains and the total score of the AQLQ-S.

## Discussion

Overall, we observed mild quality of life impairment in our sample of adult patients with asthma recruited in Southern England, as measured by the mini version of the Juniper Asthma Quality of Life Questionnaire (AQLQ-J) and the Marks Asthma Quality of Life Questionnaire (AQLQ-S). Like in a previous Dutch study [13], this may be explained by the fact that all patients were recruited through primary care.

Missing item-level data were imputed as the mean of at least 50% of the subscale items. Ordinarily this might be expected to reduce the precision of calculated statistics. However, in this context imputation of values from at least 50% of subscale items is acceptable, since the subscale items were highly correlated with each other (as should be expected for items on a common subscale). Hence it was reasonable to infer a given missing item value from the mean value of the completed items. In fact, the highest percentage of missing data among all the scales used was 9.6% for the Asthma Bother Profile.

We found acceptable internal consistency (α > 0.7) for both asthma-specific quality of life measures at total and domain level, except for the ‘environmental stimuli’ domain of the AQLQ-J.

Both measures correlated strongly with each other at the total score level. This was also true for most domain-domain correlations. Similarly strong domain-domain and domain-total as well as total-total correlations were found in a Spanish study which compared the performance of the full version of the AQLQ-J and the St George’s Respiratory Questionnaire (SGRQ) [27].

Correlations with the symptom score, particularly the asthma module of the symptom score, were strong for the AQLQ-J and moderate for the AQLQ-S.

The strength of the correlation of the AQLQ-J (*rho* = 0.69) with asthma symptoms we found was comparable to that found in the Dutch study (*rho* = 0.65) [13]. Taking the perspective of van der Molen et al. [13] this may be interpreted as evidence for (cross-sectional) construct validity, although we have taken the stance before that not symptoms per se, but the impact of symptoms is influencing asthma-specific quality of life [6]. Hence, the strong correlation of the AQLQ-J with the symptom score may raise the question of overlap between the symptom score construct and the quality of life construct as operationalized in the AQLQ-J.

The stronger correlation of the AQLQ-J compared to the AQLQ-S with the symptom score seems plausible in the light of the fact that the AQLQ-J has more symptom-related items compared to the AQLQ-S.

The finding that correlations of both measures (at total and domain level) with lung function measures were weak, if not absent, was expected. It corroborates previous findings [13,14].

The AQLQ-J showed more and stronger correlations with lung function measures. This was expected because the AQLQ-J has more symptom-based items. The findings for lung function contrasted with the strong correlations found for asthma bother and asthma control. Taking the moderate strength of the correlations with generic quality of life into account, we interpret the findings from these correlational analyses as supporting evidence for construct validity of both measures.

The relationships of the AQLQ-J and AQLQ-S with health care utilization as measured by GP visits and with perceived asthma severity were as expected and support the discriminative ability of both measures. The observed gender difference is less clear. There are few studies looking into gender differences in asthma-specific quality of life. A very recent study from India found that women reported poorer quality of life, especially in the symptoms and emotional domains of the AQLQ [28]. The findings point in a similar direction to our findings, however we found significant differences in the “environmental stimuli” and “activity limitation” domains.

## Conclusions

This study provides evidence for the validity of the mini AQLQ-J and the AQLQ-S in a British population of adults with asthma who are managed in primary care. The correlations with lung function and symptoms were different for the two measures and stronger for the mini AQLQ-J compared to the AQLQ-S, reflecting partly the different content of the questionnaires. Both the mini AQLQ-J and the AQLQ-S are able to discriminate between patients with differing levels of asthma severity.

## Competing interests

The author(s) declare that they have no competing interests.

## Authors’ contributions

CA conceived the study presented, carried out the statistical analyses, coordinated discussion between the study team and wrote the manuscript. CJ played a major role in data acquisition in the study from which the data was drawn, helped in the interpretation of the data and revised the manuscript critically for important intellectual content. MH helped with data analysis and interpretation of the data, and revised the manuscript for important intellectual content. HS conceived the study from which the data was drawn, participated in the design, coordination and manuscript preparation for this study. She revised the manuscript critically for important intellectual content. All authors read and approved the final manuscript.
